# Combined Albendazole and Praziquantel Therapy in an Adult Female with Neurocysticercosis and Generalized Tonic-clonic Seizures

**DOI:** 10.7759/cureus.2996

**Published:** 2018-07-18

**Authors:** Katerina Petrov, Faith Ihongbe, May Chang, Sarfraz Choudhary, Deepak Bhatia

**Affiliations:** 1 Department of Pharmacy Practice, Shenandoah University - Inova Center for Personalized Health Fairfax/Bernard J. Dunn School of Pharmacy, Fairfax, USA; 2 Department of Pharmacy, Inova Alexandria Hospital, Alexandria, USA; 3 Department of Pharmacy, Inova Loudoun Hospital, Leesburg, USA; 4 Department of Internal Medicine Infectious Diseases, Inova Loudon Hospital, Leesburg, USA; 5 Department of Pharmacogenomics, Shenandoah University - Inova Center for Personalized Health Icph) Fairfax Campus/Bernard J. Dunn School of Pharmacy, Ashburn, USA

**Keywords:** neurocysticercosis, albendazole, praziquantel, seizure

## Abstract

The use of albendazole monotherapy has been favored as the treatment option for neurocysticercosis. This case reports an incidence of neurocysticercosis in a 32-year-old woman who presented to the emergency department with a generalized tonic-clonic seizure. Neurocysticercosis was confirmed by magnetic resonance imaging (MRI) of the brain and a positive Taenia solium serologic test. The patient was treated with the combined dual therapy of albendazole and praziquantel at standard doses for a minimum effective duration of 14 days in the setting of scarce patient resources. She tolerated therapy without any adverse reactions and remained seizure free. A repeat MRI scan post-treatment revealed the complete eradication of cysticerci. Clinicians should be aware of and consider the efficacy and safety of combined albendazole and praziquantel therapy for the treatment of neurocysticercosis.

## Introduction

Neurocysticercosis (NCC) is a parasitic infection of the nervous system that is caused by an infection of the brain and its membranes by the larval stage of the tapeworm Taenia solium. It is a major cause of acquired epilepsy worldwide [1] and the most common cause of human parasitic neurological disease in Africa, Asia, and Latin America. It is spread mainly through contaminated food and water via the fecal-oral route [2]. Embryonated eggs and/or gravid proglottids in contaminated food or water supply ingested by humans hatch and release oncospheres. These oncospheres penetrate the intestinal wall and circulate to several body tissues, showing strong tropism for the central nervous system, where they develop into larvae (cysticerci), causing neurocysticercosis. In 2016, the Centers for Disease Control and Prevention (CDC) categorized NCC as one of the “neglected parasitic infections,” which is part of a group of five parasitic diseases in the US targeted at public health intervention [2]. Although NCC is rare in the US, it is becoming more prevalent as the rate of immigration and travel is fast increasing. The CDC reports an average of 2,300 hospitalizations annually due to NCC, with an inpatient mortality rate of 1.4% [2]. It is of increasing importance that healthcare professionals are aware of the rising prevalence of NCC and know how to prevent and effectively treat it.

Current American Academy of Neurology guidelines recommend oral albendazole (ABZ) monotherapy at 400 mg twice daily for 30 days in conjunction with oral dexamethasone or prednisone to treat NCC [3]. Alternative therapy is oral praziquantel (PZQ) monotherapy used off-label at 1,200 mg three times daily for 14 days. However, the above regimens achieve complete cysts eradication in only 30% to 40% of patients [4]. Combined therapy of ABZ and PZQ at standard recommended doses for 21 days has been suggested to be more effective in reducing viable cysticerci [5]. The two drugs have different mechanisms of action and work synergistically to give better cysticerci killing than ABZ or PZQ monotherapy alone [6].

## Case presentation

A 32-year-old woman (59 kg, 1.68 m) with a five-year history of generalized tonic-clonic seizures presented to Inova Loudoun Hospital's emergency room on June 6, 2016. Upon presentation, the patient suffered a generalized tonic-clonic seizure that lasted for more than one minute. She recently immigrated from Honduras, had no US medical insurance, and reported a history of nonadherence to her antiepilepsy medications. Currently, the patients were not on any routine home medications as an outpatient and denied any known treatment for NCC. The patient admitted that she had a seizure episode in Honduras three years ago, which was treated with intravenous (IV) fosphenytoin at the time. She had been seizure-free since.

In the emergency department, a computed tomography (CT) scan of the brain was performed without remarkable findings. The patient was loaded with a single dose of IV levetiracetam 1000 mg and then started on oral levetiracetam 500 mg twice a day after admission to the hospital. Given her prior seizure history, a magnetic resonance imaging (MRI) scan of the brain was requested to rule out any space-occupying vascular or ischemic insult. An electroencephalogram (EEG) was also requested. The patient was afebrile with mild leucocytosis (WBC 11.02) and elevated creatinine kinase (CK) of 1.6. The liver panel was normal. The physical exam and review of systems were noncontributory.

On hospital day 2, the MRI scan of the brain revealed a 5 mm ring-enhancing lesion in the posterior right frontal lobe of the cerebral cortex, with surrounding vasogenic edema, suggestive of an infective neurocysticercosis lesion. CT scan and EEG were normal. A positive serological antibody test utilizing a western blot assay for cysticercosis immunoglobulin G (IgG) antibody established the diagnosis of parenchymal neurocysticercosis. The patient was commenced on oral ABZ 400 mg twice daily plus oral PZQ 1,200 mg three times daily, dexamethasone 6 mg IV daily, and oral levetiracetam 500 mg twice daily.

On hospital day 3, no recurrent seizure episodes were observed. Leucocytosis and elevated CK resolved. On hospital day 4, the patient was discharged and given further instructions to continue taking oral ABZ 400 mg twice daily in combination with oral PZQ 1,200 mg three times daily for a total of 14 days, oral dexamethasone 6 mg daily for a total of 14 days, and oral levetiracetam 500 mg twice daily for one year. Follow-up appointments at the neurology clinic were scheduled on a monthly basis to evaluate the resolution of neurocysticercosis cysts, the presence of any seizure episodes, and other associated symptoms. The first follow-up visit in three weeks revealed no recurrent seizure episodes. The patient had finished and tolerated well the 14-day course of combination ABZ plus PZQ. Physical exam and labs were normal. A repeat MRI scan of the brain confirmed the complete resolution of cysticerci. The patient continues to be seizure free and is being monitored for one year.

## Discussion

This case report describes the successful trial of using a shorter 14-day course of ABZ and PZQ combination therapy in treating neurocysticercosis rather than a 21-day course of ABZ and PZQ combination therapy [7]. The combination therapy at standard recommended doses was safe, without any observed adverse effects. The shorter treatment duration of 14 days was discussed and decided on due to the patient's history of medication non-adherence, the potential of adverse medication side effects, and in light of the patient's limited resources for affording the cost of treatment. The patient was successfully treated, as evidenced by the complete eradication of cysticerci in the brain and the resolution of associated symptoms. A concurrent 14-day course of corticosteroid therapy was administered in addition to anthelmintic treatment to reduce the inflammatory response in the brain in conjunction with a year-long anticonvulsant therapy to prevent any recurrent seizures.

## Conclusions

This case demonstrates the treatment efficacy of a 14-day course of combination ABZ plus PZQ at standard recommended doses. Clinicians should be aware of the increasing prevalence of NCC and consider the efficacy and safety of a 14-day combined therapy of ABZ and PZQ for the treatment of neurocysticercosis. The short treatment duration of 14 days may be helpful in the setting of scarce resources, limited access to healthcare and non-adherence to medications.

## References

[1] Garcia HH, Del Brutto OH (2005). Neurocysticercosis: updated concepts about an old disease. Lancet.

[2] Parasites—cysticercosis C. Atlanta, GA GA (2018). Parasites - Cysticercosis. https://www.cdc.gov/parasites/cysticercosis/index.html.

[3] Baird RA, Wiebe S, Zunt JR (2013). Evidence-based guideline: treatment of parenchymal neurocysticercosis: report of the Guideline Development Subcommittee of the American Academy of Neurology. Neurology.

[4] Sotelo J, Del Brutto O, Penagos P, Escobedo F, Torres B, Rodriguez J, Rubio F (1990). Comparison of therapeutic regimen of anticysticercal drugs for parenchymal brain cysticercosis. J Neurol.

[5] Garcia HH, Lescano HG, Gonzalez I (2016). Cysticidal efficacy of combined treatment with praziquantel and albendazole for parenchymal brain cysticercosis. Clin Infect Dis.

[6] Harnett W (1988). The anthelmintic action of praziquantel. Parasitol Today.

[7] Petrov K, Ihongbe F, Choudhary S (2016). Case report of a combined albendazole and praziquantel therapy in an adult female with neurocysticercosis and generalized tonic-clonic seizures. Pharmacotherapy.

